# Classification of Self-Driven Mental Tasks from Whole-Brain Activity Patterns

**DOI:** 10.1371/journal.pone.0097296

**Published:** 2014-05-13

**Authors:** Norberto Eiji Nawa, Hiroshi Ando

**Affiliations:** 1 Center for Information and Neural Networks (CiNet), National Institute of Information and Communications Technology (NICT) and Osaka University, Suita, Osaka, Japan; 2 Universal Communication Research Institute, National Institute of Information and Communications Technology (NICT), Seika-cho, Soraku-gun, Kyoto, Japan; Laureate Institute for Brain Research and The University of Oklahoma, United States of America

## Abstract

During wakefulness, a constant and continuous stream of complex stimuli and self-driven thoughts permeate the human mind. Here, eleven participants were asked to count down numbers and remember negative or positive autobiographical episodes of their personal lives, for 32 seconds at a time, during which they could freely engage in the execution of those tasks. We then examined the possibility of determining from a single whole-brain functional magnetic resonance imaging scan which one of the two mental tasks each participant was performing at a given point in time. Linear support-vector machines were used to build within-participant classifiers and across-participants classifiers. The within-participant classifiers could correctly discriminate scans with an average accuracy as high as 82%, when using data from all individual voxels in the brain. These results demonstrate that it is possible to accurately classify self-driven mental tasks from whole-brain activity patterns recorded in a time interval as short as 2 seconds.

## Introduction

Functional magnetic resonance imaging (fMRI) can acquire data from thousands of different localities (voxels) of the brain at the same time. Multi-variate pattern analysis (MVPA) [1] capitalizes on that power by simultaneously looking at changes in blood-oxygenated dependent (BOLD) signal across different voxels, as opposed to the traditional univariate analysis that examines the activity of single voxels independently [2], [3]. MVPA has been used to successfully identify patterns of brain activity that characterize, for instance, responses to a certain stimulus type [4] or emotional state [5]. In this study, eleven participants were asked to engage in self-driven mental tasks - tasks that do not require attention to or processing of external stimuli - that involved counting down numbers and remembering negative and positive autobiographical episodes of their personal lives. For 32 seconds at a time, participants could solely concentrate on the execution of the tasks, with no additional experimental duties, such as attending to visual cues. We expected that these rather long time intervals would enable participants to more freely and naturally engage in the execution of the tasks. Though there have been studies that sought to classify cognitive states using data from the entire brain under well controlled conditions [6], [7], attempts to do that using experimental designs that allow participants to engage in a stream of thoughts in a self-driven, self-paced and more naturalistic manner are still rare [8].

Typically, the first step in studies applying MVPA is feature selection [9], in which a subset of voxels in the brain is selected to be considered in the analysis. Feature selection is a sensitive choice when there are well-defined hypotheses or regions of interest beforehand [10] but in the absence of those, or if it is reasonable to expect that information is represented in larger areas of the brain, it may not be a necessary step for successful classification [8], [10], [11]. In this study, because participants were performing relatively complex tasks, we hypothesized that the cognitive processes underlying the execution of those tasks would be better characterized by taking into account the activity in the entire brain rather than looking at any single region or subset of voxels. We believe this to be true for the great majority of higher-level cognitive processes and emotional states [12] as well. Moreover, because participants had considerably more behavioral freedom than in typical experiments, and given the notoriously noisy nature of the BOLD signal, we expected that there would be much less consistent activation at the single voxel level within and across participants, potentially making it much more challenging to describe the underlying neural processes if assuming a priori the existence of a set of functionally localized, discrete relevant regions. Differently from studies that averaged BOLD signal over several scans to enhance the signal-to-noise ratio (SNR) [6], [13], [14], we asked whether whole-brain activity patterns contained in a single fMRI scan, without prior selection of features, could be used to determine which mental task a participant was performing at a given point in time.

## Materials and Methods

### Participants

This study was approved by the Ethics and Safety Committees of the National Institute of Information and Communications Technology and ATR Institute International, where the experiments were conducted. We recruited 11 healthy right-handed and native speakers of Japanese (21–37 years; 5 males) using the services of a part-time employment agency. All participants signed informed consent before participation and were remunerated for their time.

### Task Instructions

Participants performed three types of mental tasks in this study following the instructions described below (the overall experimental paradigm is explained in the next section):

- Countdown task: participants were asked to start counting down from 100 at a comfortable pace (such as one subtraction every second), as soon as they were cued. They were told not to worry about occasional mistakes, and to keep counting until they heard the auditory cue signaling them to stop. If they reached 0 during the execution of the task, they were instructed to start over again from 100.
- Positive Autobiographical Memory task (PAM): approximately two weeks before the scheduled date of the study, we asked participants to prepare a list with at least 5 positive events that they had experienced in their personal lives. Participants were told that these events did not necessarily have to be important landmarks in their lives; they were free to include more mundane life events in the list as long as they were genuinely fond of reminiscing about them. To illustrate examples of positive events, we told participants that such events should involve people, places or things that they liked and enjoyed, and should be associated with feelings of happiness, satisfaction and elation. There were no restrictions regarding the contents of their memories or the recency of the events on which the memories were based. On the day of the study, participants were asked to select items in the list to remember during the experiment. We asked them to focus on the circumstances that led to the event, their feelings at that moment, who they were with and other relevant aspects surrounding that event, in as much detail as possible. In addition, during the task we asked participants to maintain a constant flow of recollections associated with the event until they were cued to stop. We suggested that participants avoided switching events too often, as we thought that that could render the task unnecessarily difficult, but aside from that, participants were told that they were free to switch to a different event at their will or if they felt that would facilitate the execution of the task.
- Negative Autobiographical Memory task (NAM): likewise with the positive memories, participants were asked to prepare beforehand a list with at least 5 negative events that had occurred in their personal lives. To illustrate examples of negative events, we told participants that such events should involve people, places and things that evoked genuine feelings of sadness, gloominess and dejection. Similarly with the positive memories, no constraints were imposed on the contents of the memories; moreover, the events that had originated them could be from the recent or the more remote past. On the day of the study, participants were instructed to choose items from the list of negative events to remember during the experiment, focusing on the details of that particular experience, the circumstances that led to that event, and the feelings that were evoked at that time. Apart from the valence of the memories, in all other respects the instructions given in this task were the same as the ones used in the positive autobiographical memory task.

### Experimental Paradigm and Procedure

All participants took part in 12 experimental sessions. In 6 sessions (Type A), participants alternated between the Countdown task (32 s) and the Negative Autobiographical Memory task (32 s), with 16-second rest periods interleaved between them; this cycle was repeated 3 times in each session. In the remaining sessions (Type B), participants alternated between the Countdown task (32 s) and the Positive Autobiographical Memory task (32 s), with 16-second rest periods interleaved between them; this cycle was repeated 3 times in each session. Experimental sessions alternated between Type A and Type B; five participants started with Type A, whereas the remaining participants started with Type B.

Auditory cues marked the start and the end of each task; the cue to start the Countdown task was *“One”* (male voice, duration 398 ms), while the cues for the Negative and Positive Autobiographical Memory tasks were *“Two”* (male voice, duration 398 ms) and *“Three”* (male voice, duration 398 ms), respectively, for participants whose first session was a Type A (the cues for the Negative and Positive Autobiographical Memory tasks were reversed for the participants whose first session was a Type B). The cue to stop the task, thus indicating the start of the rest period, was a pure tone (440 Hz, duration 398 ms). Participants were instructed about the meaning of each cue before entering the scanner, and reminded verbally about them at the start of each session. No specific instructions were given regarding what they were expected to do during the rest periods other than advising them to use that time to rest and relax. Participants were asked to immediately acknowledge the auditory cues by pressing a button, and immediately begin (or halt) the execution of the task. Only blocks in which participants acknowledged the task-start cue within 2000 ms were considered in the subsequent analyses. Experiments were performed in a darkened room, and participants were asked to keep their eyes closed during scanning. Participants had the choice to have breaks in between sessions, if they felt tired or needed time to regain concentration. Most participants occasionally chose to have 1-minute breaks over the course of the experiment. Once outside the scanner, participants filled out a questionnaire with questions regarding the experiment, such as task difficulty and task performance. They were asked to describe the life events that originated the memories remembered during the experiment, including how much time had passed since their occurrences. Using a numerical scale (0: low to 10: high), participants were also asked to rate the *pleasantness* of the memories retrieved during the PAM, the *unpleasantness* of the memories retrieved during the NAM, as well as the vividness of the respective memories. Experimental sessions lasted approximately 90 minutes, including preparation and debriefing time.

### Data Acquisition

Brain imaging data was acquired using a 3T Siemens Magnetom Trio, A Tim System scanner (Siemens Healthcare, Erlangen, Germany) equipped with a 12-channel standard head coil. Participants lay supine in the scanner and wore padded headphones, from where instructions and auditory cues were delivered. Behavioral responses (right index finger) were given via a MRI-compatible response pad connected to a computer that logged reaction times. The same computer ran a program written in Presentation (Neurobehavioral Systems, Inc., Albany, CA) that controlled the delivery of the auditory cues by time-locking it with the acquisition of functional images. Head motion was minimized using foam padding on the sides of the head. Right before the start of the experimental sessions, T2-weighted anatomical images were acquired in the same plane as the functional images using a turbo spin echo sequence (TR = 6000 ms, TE = 57 ms, FA = 90°, FOV = 192×192 mm, matrix size = 256×256, in-plane resolution 0.75×0.75 mm). In each experimental session, 147 whole-brain echo-planar functional images were acquired in 33 contiguous 4 mm axial slices parallel to the AC-PC line (TR = 2000 ms, TE = 30 ms, FA = 80°, FOV = 192×192 mm, matrix size = 64×64, in-plane resolution 3×3 mm). Before analyses, the first 3 scans of each session were discarded to account for magnetic saturation effects. Whole-brain T1-weighted anatomical images (1 mm^3^) were also acquired on a different day prior to the experiments.

To minimize the effects of physiological noise in the imaging data, cardiac and respiratory data were recorded during scanning (AD Instruments, Dunedin, New Zealand). Cardiac data was monitored using a piezoelectric pulse transducer attached to the index finger of the participant’s left hand, while respiration was monitored using a transducer belt strapped around the upper abdomen. The sampling rate of both signals was 1 kHz; the trigger signal output by the scanner at the start of each functional image acquisition was recorded to allow temporal registration of the physiological data to the imaging data.

### Data Preprocessing

Cardiac and respiratory waveforms were visually inspected to confirm that there were no major problems with the measurements. In-house routines written in Matlab (version R2007a, Mathworks, Inc., Natick, MA) were used to determine the cardiac trigger times from the waveforms. RETROICOR [15] was applied to the imaging data to reduce the effects of cardiac and respiratory cycles. In addition, clean-up techniques based on estimated respiratory [16] and cardiac [17] response functions were employed to regress low-frequency BOLD fluctuations due to variations in breathing and heart rates. The resulting functional scans were then preprocessed using SPM 5 (Wellcome Trust Centre for Neuroimaging, UK, http://www.fil.ion.ucl.ac.uk/spm/software/spm5): slice timing correction was performed using the first slice as a reference, followed by realignment and adjustment of head motion using the first image of each session as a reference, after realigning the first image of each session to the first image of the first session (no subjects moved more than the length of a voxel in any one direction, thus none were excluded from the analysis); functional and anatomical images were co-registered using a two-step procedure involving the participant’s T2- and T1-weighted anatomical images. Functional images were spatially normalized to the standard stereotaxic Montreal Neurological Institute (MNI) space by applying the transformation matrix derived by normalizing the T1-weighted anatomical image to the SPM 5 *templates/T1.nii* image. The original voxel size was kept the same throughout these steps (3×3×4 mm) resulting in images of size (53, 63, 35) voxels in the (X, Y, Z) dimensions, respectively, after normalization. From here, two different processing pipelines were used to prepare the data for the general linear model (GLM) analysis, and the machine learning based analysis. Scans used in the GLM analysis were spatially smoothed using a Gaussian kernel of 8-mm full width at half maximum (FWHM). For the data used in the machine learning based analysis, there were four additional steps after the spatial normalization. First, nuisance variables were regressed from the BOLD time series of each voxel: the six affine head motion parameters estimated during the realignment step, the mean time series of a region corresponding to white matter (3-mm sphere centered at MNI coordinates *x = *26, *y = −*12, *z = *35), and cerebrospinal fluid (CSF) (3-mm sphere centered at MNI coordinates *x = *19, *y* = −33, *z = *18), the mean time series across the whole-brain (global signal), computed by using a binary mask generated by thresholding the SPM 5 image *apriori/grey.nii* at 0.22, plus a constant regressor for each one of the sessions to account for the mean session effect. Next, the BOLD time series of each voxel was high-pass filtered (cut-off frequency of 0.008 Hz), and the voxel values recorded within a session were scaled to a grand mean of 100. Finally, the BOLD time series of each voxel was standardized by subtracting the mean and dividing it by the standard deviation, with both values computed from the time series of the respective voxel over the entire experiment.

### General Linear Model Analysis

At the individual-level, brain activity during the execution of mental tasks was estimated on a voxel-by-voxel basis using the GLM implemented in SPM 5. The time series for each voxel was high-pass filtered to 0.0078 Hz, and serial correlations were corrected by an autoregressive AR (1) model.

The GLM had two regressors of interest that corresponded to the two mental tasks performed in each session: Countdown and Negative Autobiographical Memory tasks (Type A) or Countdown and Positive Autobiographical Memory tasks (Type B). Regressors of no interest were the six parameters describing head motion plus the constant regressors accounting for the mean session effect. The brain activity elicited during the execution of the tasks was modeled by a boxcar function of the duration of the task (32 s) at the onset times of the acknowledged blocks, convolved with the canonical hemodynamic response function provided in SPM 5. Linear contrast images were generated for each participant using pairwise comparisons between tasks or between the task and the implicit baseline. The participant-specific contrast images of parameter estimates were used as inputs to a random effects model to permit group-level inferences [18]. The resulting statistical maps were submitted to a voxel-level threshold of *p*<.005 uncorrected and a cluster extent threshold of *p<*.05 corrected for the whole brain. The cluster extent thresholds were determined for each group-level analysis using the function CorrClusTh.m (v.1.12) written in Matlab by Thomas Nichols, and were in the range of 78 to 98 voxels.

### Machine Learning Based Scan Classification

The 116 spatial masks in the Automated Anatomical Labeling library (AAL) [19] were used to determine the voxels to be used in the analysis. The masks covered cortical regions, subcortical structures and the cerebellum. BOLD time-series from these voxels were extracted from the functional images (scans) of each participant using MarsBaR [20]. There were 40,761 voxels in the comprehensive mask formed by the 116 regions (voxel size of 3×3×4 mm). Note that by using such a mask, the number of voxels effectively used to perform the classification is reduced to approximately 34.9% of the original number (116,865). In fact, the number of voxels from where signal could be actually extracted varied across participants (range of 40,068 to 40,761 voxels, with median 40,688), possibly due to small discrepancies in shape that remained even after the spatial normalization. No feature selection [9] was performed in the data: each scan was encoded as an array of size *D_k_*, where *D_k_* is the number of voxels in the image resulting from the conjunction between the comprehensive mask and the functional images of participant *k*. This amounts to saying that under this representation scheme, the data unit manipulated by the classifiers is a “snapshot” of the entire brain consisting of the BOLD signal value of all voxels recorded in a time interval of TR = 2 seconds.

Machine learning based classifiers were trained and tested using the whole-brain functional images acquired during the task blocks. Each task block consisted of 16 scans; because there were 3 blocks of each task type in each session, if the participant correctly responded to all start-task auditory cues in the experiment, there would be 576 scans for the Countdown task and 288 scans for each Memory task (Negative and Positive). We used linear support-vector machines (SVMs) to train the classifiers [21], using the implementation available in the library LIBSVM version 3.11 [22]. The parameter *C* [21], which determines the penalty on misclassified data points, was set to 1 in all runs. The classifiers were tested on their accuracy of determining which mental task – out of two possible alternatives – a participant was performing during the acquisition of individual functional images. Classification was performed using scans acquired in sessions of the same type (Countdown vs. Negative Autobiographical Memory and Countdown vs. Positive Autobiographical Memory), and also between scans acquired in different sessions (Negative Autobiographical Memory vs. Positive Autobiographical Memory). Classification accuracy was examined using a leave-one-session-out cross validation scheme: scans from one of the sessions were put aside to assess the classifier’s accuracy after training (test dataset), while scans from the remaining sessions of the same type were used to train the classifier (training dataset). This procedure was repeated so that data from every session served once as a test dataset. Before training, values in the training dataset were scaled to the interval [–1, 1]. The same scaling parameters, i.e., the maximum and minimum of each time course in the training dataset, were applied to scale the test dataset before assessing the classifier’s accuracy.

Because the Negative and Positive Autobiographical Memory tasks were not performed in the same session, when performing the pairwise classification of the Negative vs. Positive Autobiographical Memory scans, the test dataset in each iteration of the cross-validation contained scans from two adjacent sessions, i.e., sessions 1–2, 3–4, etc.

Datasets were strictly balanced by ensuring that the number of scans in each class was the same; if a behavioral response was not registered at the start of a task block, e.g., the 2^nd^ block of the Negative Autobiographical Memory task, the scans from that block and the scans from the equivalent block of the counterpart task, e.g., the 2^nd^ block of the Countdown task in the same session, or the 2^nd^ block of the Positive Autobiographical Memory task in the paired session, were excluded from the dataset, regardless of whether a response was given or not. Note that as a result of this additional screening, for a few subjects the data used in the scan classification analysis was a subset of the data used in the GLM analysis. Classification accuracy was the proportion of correctly classified scans (*c*) from the total number of valid scans remaining after the screening (*n*). Note that *c* is computed by pooling the results of all iterations of the cross-validation. Statistical significance was examined using a balanced-block permutation test [23], that differently from an ordinary permutation test, maintains the block structure of the experiment when shuffling the labels to account for the sluggishness of the hemodynamic response. In addition, to provide a measure of precision, the 95% confidence interval was computed based on a beta distribution Beta(*c+*1, *e+*1), where *e* was the total number of incorrectly classified scans [24], [25].

In order to verify the effects of using a coarser representation of brain states, we performed the exact same analysis described above using the average BOLD signal value over the voxels in each one of 116 anatomical regions defined by the AAL, instead of using the individual voxel values. Under this representation scheme, each scan was encoded as a vector of size *D_k_* = 116. Classifiers were trained and tested using the same cross-validation and data balancing schemes as before.

Classification accuracy in the across-participants analysis was examined using a leave-one-subject-out cross-validation scheme: at each iteration, data from one participant was tested on classifiers that were trained using the data from the remaining participants, and this was repeated until scans from all participants were used to test the classifiers. The number of scans in each class was balanced at the individual level, therefore, each participant contributed an equal number of scans from each class. However, that number was not the same across participants due to differences in the number of task blocks each participant acknowledged during the experiments. Classification accuracy, its significance and precision were computed as in the within-participant classification analysis. Here again, we performed the analysis using the voxel-based and the region-based representations. Because the number of voxels covered by the whole-brain images differed across participants, only the 39,799 voxels that were common to all participants were taken into consideration in the across-participants analysis.

## Results

### Behavioral Results

The majority of the participants diligently responded to all auditory cues signaling the start of a task block. One participant missed 1 cue, while two other participants missed 4 of the 72 start cues delivered during the whole experiment. All other participants had a perfect record. There were no significant differences of average reaction time for the start-task cue across task types (F(2, 20) = 3.216, *p* = .0615, repeated measures ANOVA). The debriefing data showed that in average participants remembered 1.8 different events in both the Negative Autobiographical Memory task (range of 1 to 6), and the Positive Autobiographical Memory task (range of 1 to 5). Using a scale of 0: low to 10: high, the average *unpleasantness* of the negative memories was 7.3 with standard deviation (SD) 2.9, while their average *vividness* was 7.5 (SD = 1.4) using the same scale. The average *pleasantness* of the positive memories was 7.6 (SD = 2.2), and their average *vividness* was 6.8 (SD = 2.1), both using the same scale. (Table S1 lists a summary of the events recollected during the experiments, as reported by the participants at debriefing time.).

### GLM Analysis Results

In the GLM group-level analysis, we first examined whether there were voxels where increased BOLD signal was observed during the execution of each one of the three mental tasks (Countdown, Negative Autobiographical Memory, Positive Autobiographical Memory) compared with the implicit baseline. The MNI coordinates of the peak voxels of the clusters that survived the threshold, the values of the T-statistic and the cluster sizes, and the names of the regions where the peak voxels were located (according to the AAL library) are listed in Table 1. We then examined the pairwise contrasts between mental tasks (Table 2). The contrasts between Countdown versus the Baseline, and against each one of the Memory tasks indicated an increase in BOLD signal in voxels of the inferior parietal lobule, a region that is consistently found to be activated by numerical tasks [26]. The contrasts between Negative Autobiographical Memory versus the Baseline and the Countdown task indicated an increase in BOLD signal in voxels of, among other regions, the inferior frontal gyrus, a region that has been associated with autobiographical memory retrieval processes in past studies [27]–[29].

**Table 1:** Regions that showed greater activity at a voxel-level threshold of *p*<.005 (uncorrected) and a cluster extent threshold of *p*<.05 (corrected for the whole-brain) when performing the mental tasks compared with the implicit baseline.

Contrast	MNI coordinates	T-statistic	Cluster size (voxels)	Region name
Countdown	45, −12, 52	8.08	591	L Postcentral gyrus
vs.	–9, 0, 60	6.67	152	L SMA
Baseline	48, −15, 52	5.75	299	R Precentral gyrus
NAM vs.	42, 27, −16	11.18	123	R Inf. frontal gyrus
Baseline	–3, 60, 20	8.47	598	L Sup. medial gyrus
	–39, 24, −16	8.36	244	L Inf. frontal gyrus
	–6, −54, 20	7.38	1325	L Precuneus
	24, −75, −36	7.18	221	R Cerebellum
	9, −42, −44	4.36	115	R Cerebellum
PAM vs.	–12, −60, 12	7.5	337	L Calcarine gyrus
Baseline	12, −45, −44	6.7	120	R Cerebellum
	–9, 45, 44	5.84	166	L Sup. medial gyrus

**Table 2:** Regions with distinct activation in the direct comparison between mental tasks at a voxel-level threshold of *p<*.005 (uncorrected) and a cluster extent threshold of *p<*.05 (corrected for the whole-brain).

Contrast	MNI coordinates	T-statistic	Cluster size (voxels)	Region name
NAM vs.	–9, 57, 28	9.66	888	L Sup. medial gyrus
Countdown	21, −78, −36	8.28	2734	R Cerebellum
	–42, 24, −16	5.59	176	L Inf. frontal gyrus
	–51, −69, 24	5.4	125	L Angular gyrus
	42, 33, −12	5.09	99	R Inf. frontal gyrus
Countdown	54, −36, 52	8.97	728	R Inf. parietal lobule
vs. NAM	–51, −33, 40	8.84	607	L Inf. parietal lobule
PAM vs.	–39, −39, −4	10.63	3043	-
Countdown	–21, 33, 48	8.55	276	L Sup. frontal gyrus
Countdown	54, −36, 52	16.36	951	R Inf. parietal lobule
vs. PAM	–60, 0, 12	14.77	797	L Rolandic operculum
	45, −51, −8	6.56	174	R Inf. temporal gyrus
	–24, −60, −4	5.98	119	L Lingual gyrus
	–45, −45, 48	4.93	164	L Inf. parietal lobule
NAM vs. PAM	–9, 60, 32	5.71	129	L Sup medial gyrus
PAM vs. NAM	–	–	–	–

### Within-participant Scan Classification Results

In this analysis we examined whether the information contained in a single whole-brain functional image can be used to determine which of two mental tasks a participant is performing at a given point in time, using data from the same participant to train the classifiers. First, the voxel time series of the entire brain were extracted from the data of five sessions (or ten sessions, in the classification of memory tasks). Next, portions of the data corresponding to the blocks where the mental tasks were performed (acknowledged task blocks) were used to train a linear SVM classifier. We shifted the onsets of the task blocks by 4 s ( = 2 TRs) when extracting the data to approximately account for the hemodynamic delay [30]. Finally, the scans from the task blocks of the remaining session (or two sessions, in the classification of memory tasks) were used to test the classification accuracy of the trained linear SVM classifier. This was repeated until all sessions had been tested, and an aggregate value of the classification accuracy for that pair of mental tasks was computed. Classifiers were trained for each task pair, using both the voxel-based representation scheme (where each voxel is regarded as a feature), and the region-based representation scheme (where features are the mean values of 116 anatomical regions).

**Figure 1:**
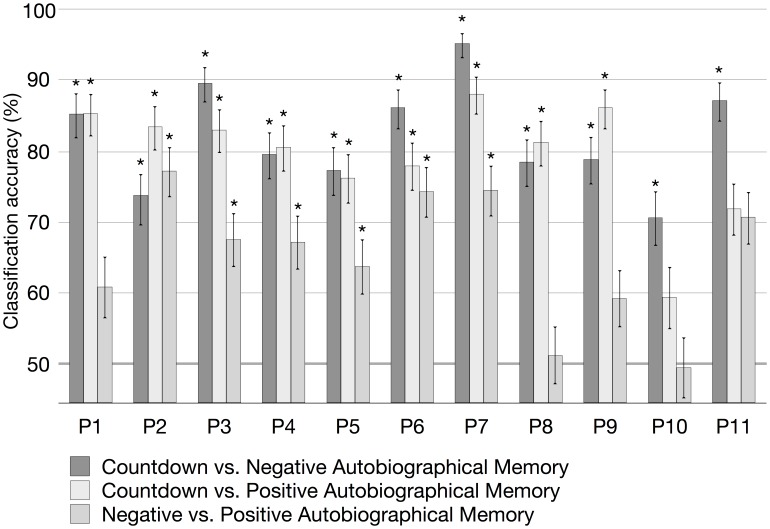
Within-participant classification accuracy in percent, using the voxel-based representation combined with linear SVMs, for each participant (P1–P11). Asterisks indicate *p<*.05 from a balanced-block permutation test, and the error bars are the 95% confidence intervals computed based on a beta distribution.

**Figure 2:**
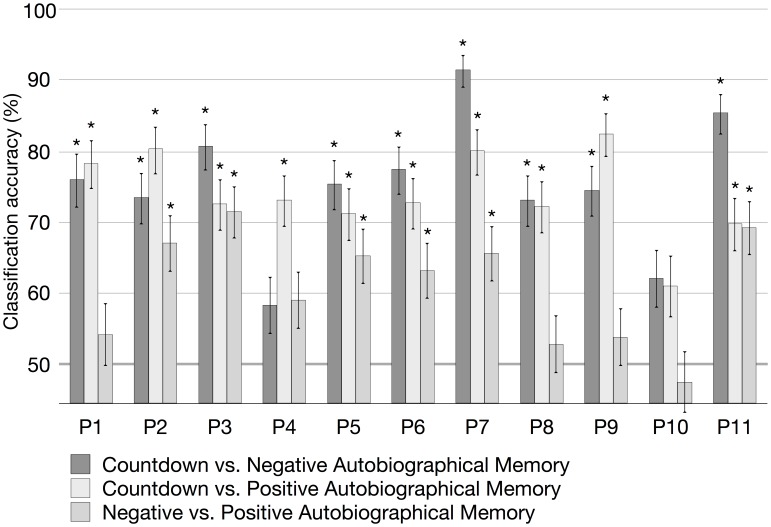
Within-participant classification accuracy in percent, using the region-based representation combined with linear SVMs, for each participant (P1–P11). Asterisks indicate *p<*.05 from a balanced-block permutation test, and the error bars are the 95% confidence intervals computed based on a beta distribution.

To verify whether the classification accuracy was above chance level, a balanced-block permutation test was conducted with 300 and 306 permutations for each participant for the classification between scans of the Countdown task and the Negative or Positive Autobiographical Memory tasks, and the classification between scans of the Negative and Positive Autobiographical Memory tasks, respectively. The 95% confidence interval was calculated for each pairwise classification based on the accumulated number of correctly and incorrectly classified scans across all iterations of the cross-validation. The individual mean classification accuracies are shown in Figures 1 and 2, for the voxel-based and region-based representations, respectively. The figures also show the results for the balanced-block permutation tests (asterisks when *p*<.05) and the 95% confidence interval (error bars).

As shown in Figures 1 and 2, linear SVM classifiers were successful in discriminating Countdown scans from Negative Memory scans significantly above chance level for all participants, using the voxel-based representation scheme (two participants failed to achieve significance when using the region-based representation scheme). Moreover, classifiers were successful in discriminating Countdown scans from Positive Memory scans significantly above chance level for all participants but two, using the voxel-based representation scheme (one participant failed to achieve significance when using the region-based representation scheme). When discriminating scans between Negative Memory and Positive Memory using the voxel-based representation, classifiers from five participants failed to achieve accuracy significantly above chance level (*n.s.*, balanced-block permutation test). When using the region-based representation, classifiers failed to correctly categorize the scans of five participants (*n.s.*, balanced-block permutation test).

**Figure 3:**
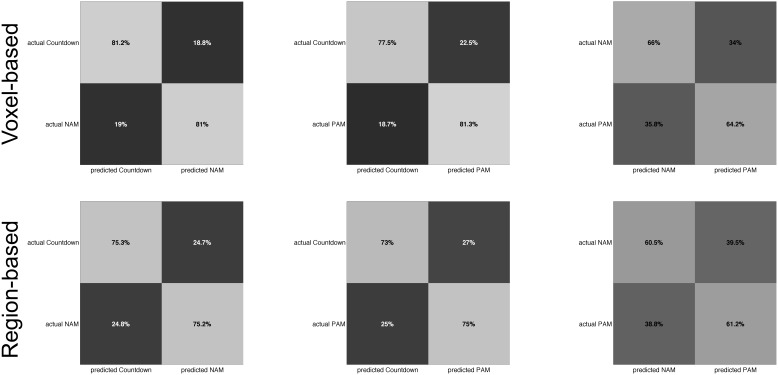
Average confusion matrices for the within-participant classifiers of each one of the task pairs using the voxel-based representation scheme (top) and the region-based representation scheme (bottom). *NAM: Negative Autobiographical Memory task; PAM: Positive Autobiographical Memory task.*

The mean and standard deviation across participants of the individual within-participant accuracies for the Countdown vs. Negative Autobiographical Memory discrimination task were 82.0%±7.3 (voxel-based representation) and 75.3%±9.3 (region-based representation). In the Countdown vs. Positive Autobiographical Memory, the mean and standard deviation were 79.4%±8.1 (voxel-based representation) and 74.0%±6.1 (region-based representation). In the classification of Memory scans (Negative vs. Positive), the mean and standard deviation were 65.1%±9.2 (voxel-based representation) and 60.8%±7.8 (region-based representation). Using the Mann-Whitney-Wilcoxon test, we verified whether the observed differences in the individual classification accuracy between the two representation schemes were significant within each participant. For all task pairs, classification accuracy was significantly higher (*p<*.05) when using the voxel-based representation scheme (*p = *.00049, Countdown vs. Negative Autobiographical Memory; *p = *.00098, Countdown vs. Positive Autobiographical Memory; *p = *.023, Negative vs. Positive Autobiographical Memory). Figure 3 shows the average confusion matrices of the within-participant classifiers for each one of the task pairs, for both the region-based and voxel-based representation schemes.

**Figure 4:**
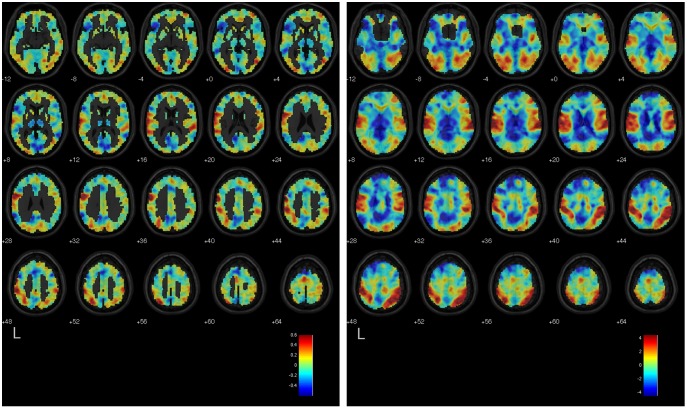
Left: Mean discriminating volume of the within-participant classifiers trained to separate scans from the Countdown and Negative Autobiographical Memory tasks. Reddish tones indicate voxels where increased BOLD signal most consistently characterized the Countdown task, as opposed to the Negative Autobiographical Memory task, according to the training results of the linear SVM classifiers. Likewise, bluish tones indicate voxels where increased BOLD signal most consistently characterized the Negative Autobiographical, as opposed to the Countdown task. Right: Unthresholded statistical map (T-statistic values) of the contrast Countdown vs. Negative Autobiographical Memory obtained from the GLM group-level analysis. Reddish tones indicate areas of the brain where increased BOLD signal was observed during the Countdown task relative to the Negative Autobiographical Memory task, and bluish tones indicate areas where the reverse effect was observed. Both maps are overlaid on a template anatomical image. Shown are axial slices from MNI *z* = −12 to 64.

**Figure 5:**
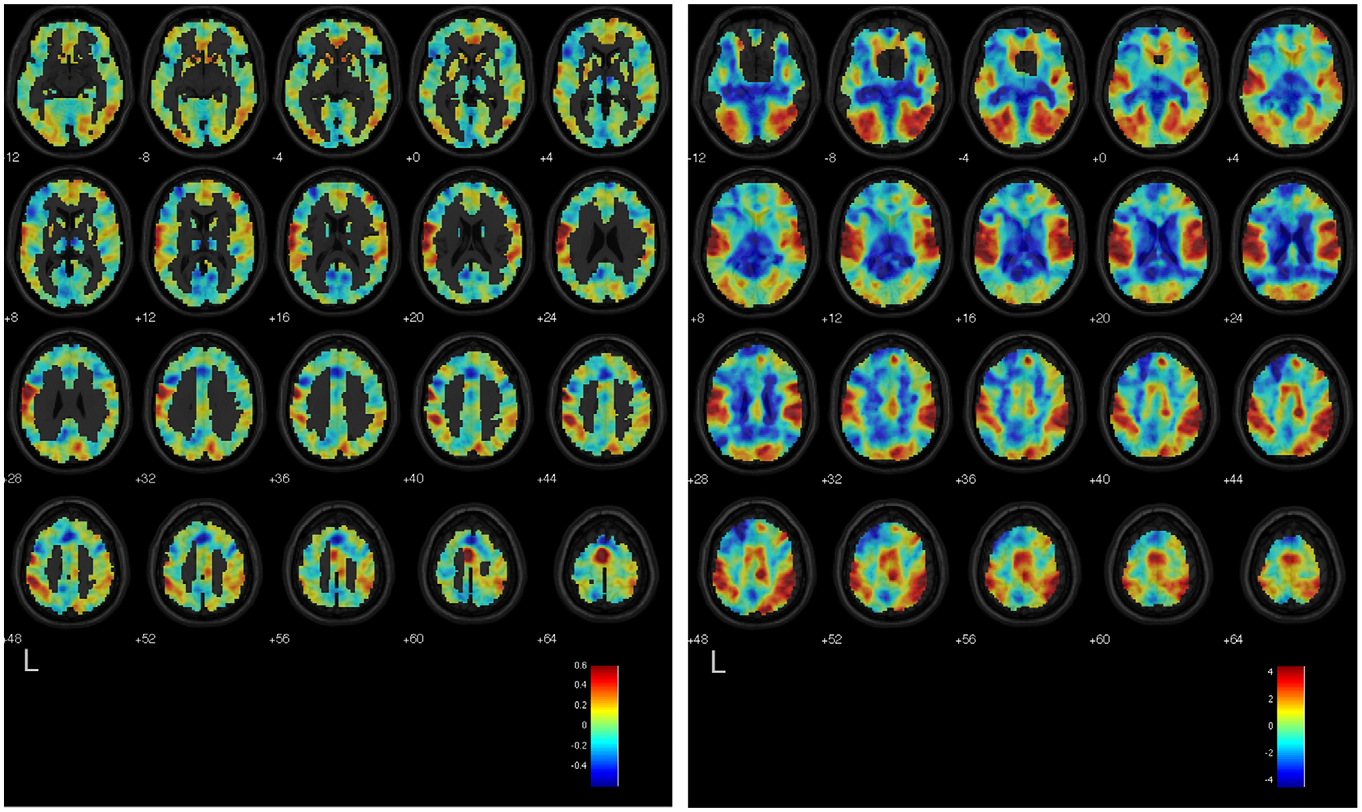
Left: Mean discriminating volume of the within-participant classifiers trained to separate scans from the Countdown and Positive Autobiographical Memory tasks. Reddish tones indicate voxels where increased BOLD signal most consistently characterized the Countdown task, as opposed to the Positive Autobiographical Memory task, according to the training results of the linear SVM classifiers. Likewise, bluish tones indicate voxels where increased BOLD signal most consistently characterized the Positive Autobiographical Memory task, as opposed to the Countdown task. Right: Unthresholded statistical map (T-statistic values) of the contrast Countdown vs. Positive Autobiographical Memory obtained from the GLM group-level analysis. Reddish tones indicate areas of the brain where increased BOLD signal was observed during the Countdown task relative to the Positive Autobiographical Memory task, and bluish tones indicate areas where the reverse effect was observed. Both maps are overlaid on a template anatomical image. Shown are axial slices from MNI *z* = −12 to 64.

**Figure 6:**
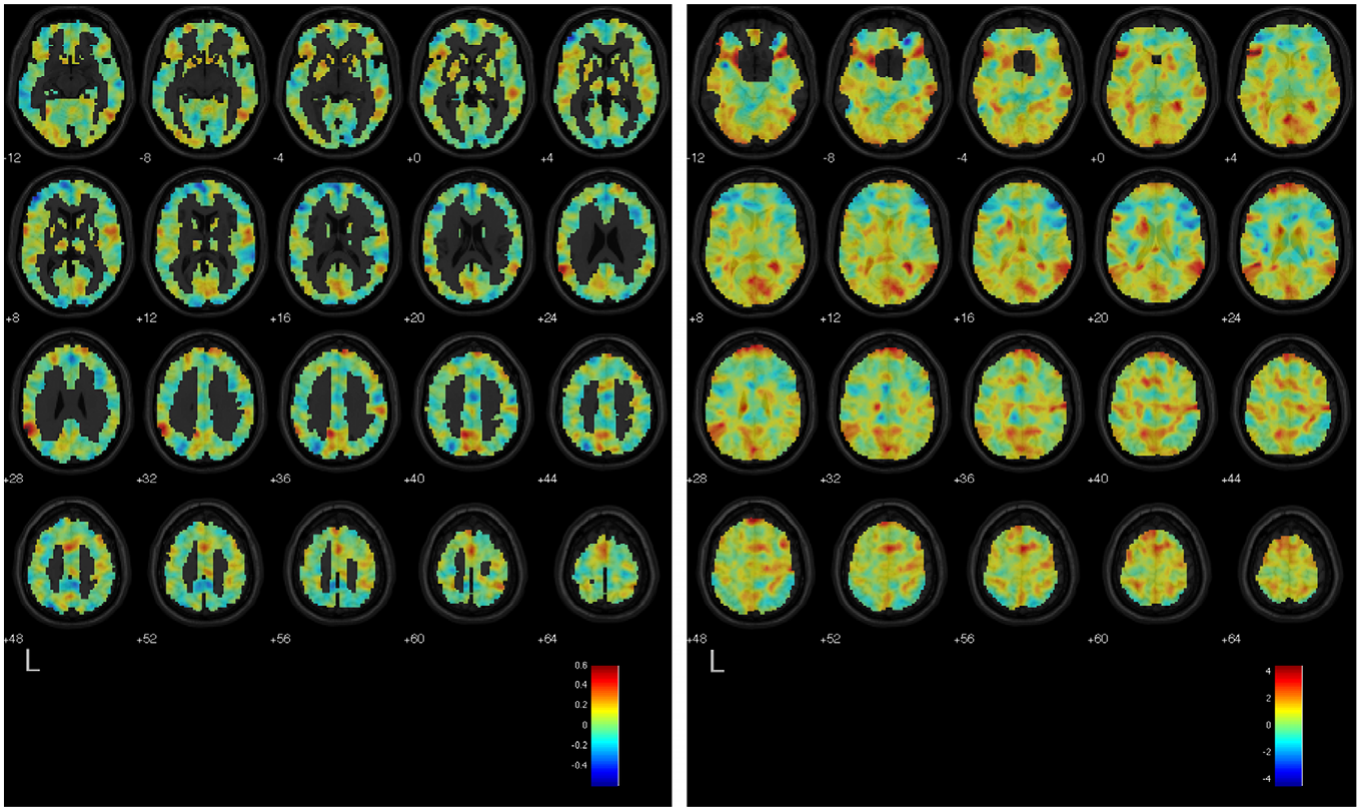
Left: Mean discriminating volume of the within-participant classifiers trained to separate scans from the Negative Autobiographical Memory and Positive Autobiographical Memory tasks. Reddish tones indicate voxels where increased BOLD signal most consistently characterized the Negative Autobiographical task, as opposed to the Positive Autobiographical Memory task, according to the training results of the linear SVM classifiers. Likewise, bluish tones indicate voxels where increased BOLD signal most consistently characterized the Positive Autobiographical task, as opposed to the Negative Autobiographical task. Right: Unthresholded statistical map (T-statistic values) of the contrast Negative Autobiographical Memory vs. Positive Autobiographical Memory obtained from the GLM group-level analysis. Reddish tones indicate areas of the brain where increased BOLD signal was observed during the Negative Autobiographical Memory task, and bluish tones indicate areas where the reverse effect was observed. Both maps are overlaid on a template anatomical image. Shown are axial slices from MNI *z* = −12 to 64.

Because we are using linear SVMs, it is possible to calculate the *discriminating volume* [7], [9] to which a classifier has converged after training, by re-projecting the results of the training back into the space of features, which in this case are the voxels or regions in the brain. The discriminating volume shows the spatial organization of the most discriminating voxels or regions, i.e., those that drive the output of the classifier when determining whether a scan belongs to the *positive class* (e.g., Countdown) or to the *negative class* (e.g., Negative Autobiographical Memory). As a general rule, voxels (regions) in the discriminating volume that have greater absolute values are more informative than voxels (regions) with lower absolute values; moreover, the sign of the voxel (region) value tells whether enhanced BOLD activity in that voxel (region), when comparing the two classes, will drive the classifier output towards the positive or the negative class. We used the results from the within-participant classifiers using the voxel-based representation scheme to calculate the mean discriminating volume over all participants for a given pair of mental tasks. We first computed each participant’s mean discriminating volume by averaging the standardized (unit-variance) discriminating volumes that resulted from each iteration of the cross-validation. We then spatially smoothed each participant’s mean discriminating volume using a Gaussian kernel of 8-mm FWHM, and averaged the resulting voxel-level values over all participants to obtain the mean discriminating volume. To measure the similarity between the results of the GLM analysis and the mean discriminating volumes, we calculated Pearson’s correlation coefficient between the T-statistic values (by voxel) in the unthresholded group-level GLM statistical maps, and the values in the mean discriminating volume elicited by the within-participant classifiers trained to discriminate the same two classes. The GLM statistical map when contrasting task 1 vs. task 2 indicates the voxels where increases in BOLD signal are associated with the execution of task 1 (positive values) or task 2 (negative values). The correlation coefficient was *r = *0.653 (*p*<.0001) for the Countdown vs. Negative Autobiographical Memory, *r* = 0.618 (*p*<.0001) for the Countdown vs. Positive Autobiographical Memory, and *r* = 0.461 (*p*<.0001) for the Negative Autobiographical Memory vs. Positive Autobiographical Memory. Figures 4, 5, and 6 show the mean discriminating volumes for the Countdown vs. Negative Autobiographical Memory, Countdown vs. Positive Autobiographical Memory, and Negative Autobiographical Memory vs. Positive Autobiographical Memory, together with the respective unthresholded statistical maps generated by the group-level GLM analysis for the contrast involving the same pair of tasks. Both data are shown overlaid on top of the SPM 5 anatomical image *single_subj_T1.nii*, limited to the axial slices from MNI *z* = −12 to 64.

**Figure 7:**
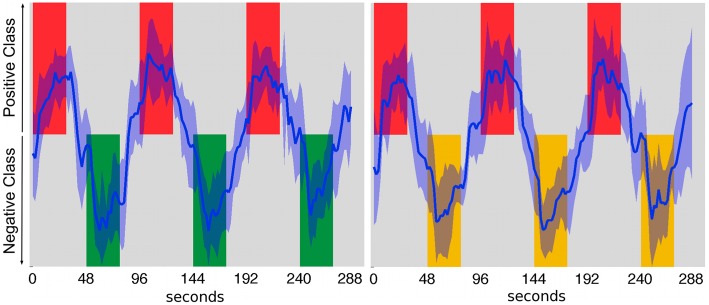
The blue lines show the time courses of the mean output over all participants of the linear SVM classifiers when trained to discriminate scans of Type A (left) or Type B (right) sessions. The horizontal axis is the time in seconds. If the output of the classifier is positive, the corresponding input (test scan) is judged to belong to the *positive class.* Likewise, if the ouput of the classifier is negative, the corresponding input is judged to belong to the *negative class.* The red blocks correspond to the scans where the participants were asked to perform the Countdown task (*positive class*), the green blocks correspond to the scans where participants were asked to perform the Negative Autobiographical Memory task (*negative class*), and the yellow blocks correspond to the scans where participants were asked to perform the Positive Autobiographical Memory task (*negative class*). The shaded area indicates the standard deviation of the outputs of the classifiers over all participants.

Figure 7 shows the time courses of the mean output over all participants of the trained linear SVM classifiers when discriminating all scans contained in a Type A (left) or Type B (right) session. The time courses include the scans corresponding to the rest periods in between task blocks, which were not used to train or test the classifiers. The output of the classifier is positive when the trained classifier judges that the input test scan belongs to the *positive class*; likewise, the output is negative when the trained classifier judges that the input test scan belongs to the *negative class*. In the analysis, the *positive class* was arbitrarily set to be the Countdown task, and the *negatives class* was either the Negative or the Positive Autobiographical Memory task (switching the assignment should not affect the results). The time courses were computed by first normalizing the values output by the classifier in each iteration of the cross-validation to the interval [–1, 1], and then calculating a mean time course for each participant. Those values were then used to calculate the mean output over all participants, which are shown in the figure.

### Across-participants Scan Classification Results

In this analysis, we examined the viability of classifying whole-brain functional images of a participant performing a mental task using a linear SVM classifier trained on data recorded from other participants. For that purpose, a leave-one-participant-out cross-validation scheme was employed to verify the classification accuracy of the trained classifiers. Data from all 11 participants were included in the training rotation, using both representation schemes, for all task pairs.

As before, the aggregate classification accuracy for each pair of mental tasks was computed as the number of correctly classified scans across all participants divided by the total number of tested scans. The significance of the classification accuracy was verified using a balanced-block permutation test with 132 permutations for the classification between scans of the Countdown task and the Negative or Positive Autobiographical Memory task, and 110 permutations for the classification between scans of the Negative and Positive Autobiographical Memory task. We also computed the 95% confidence interval based on the total number of correctly and incorrectly classified scans across all participants.

**Figure 8:**
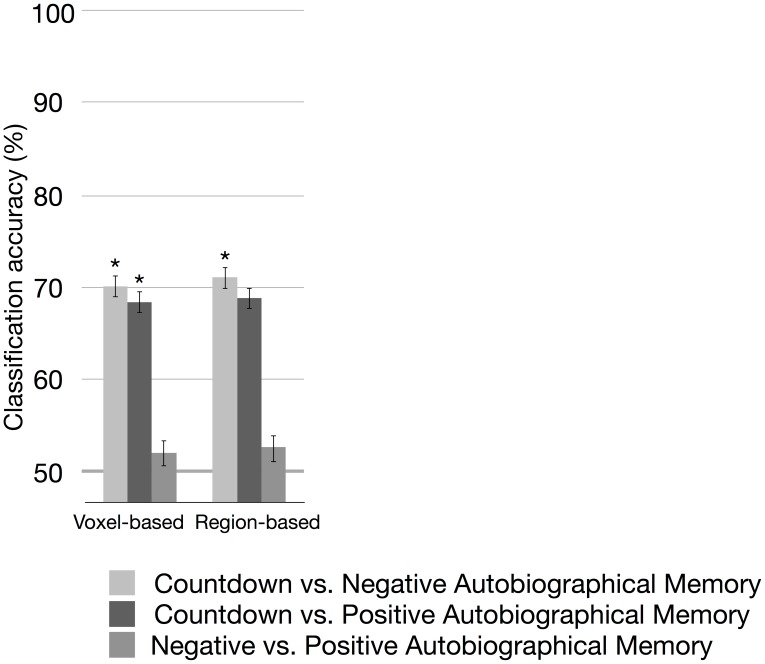
Across-participants classification accuracy in percent, using the voxel-based and the region-based representations combined with linear SVMs. Asterisks indicate *p<*.05 from a balanced-block permutation test, and the error bars are the 95% confidence intervals computed based on a beta distribution.

Using the voxel-based representation, the aggregate classification accuracy across participants for the Countdown vs. Negative Autobiographical Memory was 70.1%, which was significantly above chance (*p<*.05, balanced-block permutation test), with the 95% confidence interval ranging from 69.0 to 71.3%. For the Countdown vs. Positive Autobiographical Memory, the aggregate classification accuracy was 68.4% (*p<*.05, balanced-block permutation test), with the 95% confidence interval ranging from 67.3 to 69.6%. For the Negative vs. Positive Autobiographical Memory, the aggregate classification accuracy was 53.4% (*n.s.*, balanced-block permutation test). Using the region-based representation, the aggregate classification accuracy for the Countdown vs. Negative Autobiographical Memory was 71.1%, which was significantly above chance (*p<*.05, balanced-block permutation test), with the 95% confidence interval ranging from 69.9 to 72.2%. For the Countdown vs. Positive Autobiographical Memory, the aggregate classification accuracy was 68.9% (marginally significant at *p* = .055, balanced-block permutation test), and for the Negative vs. Positive Autobiographical Memory, the aggregate classification accuracy was 56.2% (*n.s.*, balanced-block permutation test). These results are summarized in Figure 8.

The mean and standard deviation of the individual classification accuracies, i.e., the classification accuracy attained when testing each participant’s dataset, were 70.0%±7.1 and 70.8%±9.2 for the Countdown vs. Negative Autobiographical Memory, using the voxel- and region-based representation schemes, respectively. For the Countdown vs. Positive Autobiographical Memory, the mean and standard deviation were 68.4%±7.7 and 68.7%±7.4, using the voxel- and region-based representation schemes, respectively. For the classification of Memory scans, the mean and standard deviation were 52.1%±3.3 and 52.7%±5.2, for the voxel- and region-based representation schemes, respectively. Using the Mann-Whitney-Wilcoxon test, we verified whether there were significant differences in classification accuracy by comparing the classification accuracies obtained in each round of the leave-one-subject-out cross-validation under each representation scheme. Differently from the within-participant results, in the across-participants classification no differences were found for any of the task pairs.

## Discussion

In this study, we verified the feasibility of using whole-brain fMRI data acquired in a time as short as 2 seconds to determine which mental task, out of two alternatives, a participant was performing at a given point in time, without prior selection of features. Though the classification of single brain scans has been done before ([1] and references therein), in typical studies participants have to comply with well-controlled external stimulus presentations meant to schedule, induce, or elicit specific emotional or cognitive states (for instance, [7], [31], [32]). In the current study, the experiment was organized in a blocked fashion that is common to imagery tasks [33]–[36], but within each block participants could engage in a continuous stream of thoughts in a self-driven and natural way, without having to continuously attend to external stimuli, e.g., as in [5]. Accounts of the events recollected during the experiments, according to reports obtained at debriefing time (Table S1), showed that participants engaged in remembering personal episodes that though unquestionably relevant, are much less graphically vivid than imagery strategies used by participants in other studies [5]. The current results indicate that it is possible to distinguish much more nuanced emotional states from whole-brain imaging data acquired when participants engage in a self-driven, self-paced stream of thoughts.

A similar experiment was reported in [8] involving the classification of single scans in real-time while a participant alternated between having happy and sad thoughts. However, the reported results were obtained from a single well-experienced participant, so no across-participants analysis was performed. Our study suggests that the results extend to a larger population, indicating that it is possible to achieve fairly high classification accuracy rates of single whole-brain scans in settings where participants perform relatively complex mental tasks.

The motivation of classifying whole-brain activity patterns, be they encoded using a voxel-based or a region-based representation scheme, is based on the assumption that higher-level cognitive processes are simultaneously supported by several different regions of the brain. If that is the case, the modular metaphor of brain functional organization might not be sufficient to examine, identify or classify the neural mechanisms underlying such processes. We examined a posteriori the possibility that the within-participant scan classification between the Countdown task and the Negative or Positive Memory task was being solely driven by activity patterns in the inferior parietal lobule, a region that was consistently associated with the Countdown task, relative to the Memory tasks (Table 2). The results indicate that this was not the case, showing that information relevant for classification was distributed in other areas of the brain as well (see File S1).

The presumed drawback of using whole-brain data from a machine learning perspective is that the number of features (voxels) is much higher than the typical number of samples (scans) collected in a study, leading to a problem known as the ‘curse of dimensionality’. The practical consequence is that in such cases, classifiers are usually expected to generalize very poorly. However, in theory, the generalization ability of SVMs is not bound by the number of features in the space [21], [37]. Though feature selection has been employed to implement real-time classification of fMRI scans during positive and negative imagery [5], the fairly high classification accuracies obtained in this study indicates that it may not be a required step. Moreover, reducing the number of features does not always result in improvements in classification accuracy [10], [11], at least in the context of brain imaging data.

When discriminating scans between Countdown and Negative/Positive Autobiographical Memory tasks using the voxel-based representation scheme, the mean classification accuracies of the within-participant linear SVM classifiers were in the range of 59.4 to 95.1%. These results were significantly higher than the mean classification accuracies obtained when using the region-based representation scheme (range of 58.3 to 91.5%). In the arguably much more challenging task of discriminating scans between Negative and Positive Autobiographical Memory tasks, the classification accuracies were overall lower but still the voxel-based representation scheme (range of 49.4 to 77.2%) outperformed the region-based representation scheme (range of 47.5 to 71.5%). Given that the signal-to-noise ratio (SNR) of fMRI data is notoriously low, one might have expected the exact opposite result, since averaging the signal over a group of voxels can potentially lead to a better SNR, and by moving from a voxel-based to a region-based representation scheme the number of features drops dramatically. However, these results suggest that for the purposes of distinguishing between mental tasks, the fine-grained, voxel-level BOLD activity carries relevant information for classification, which is partially lost when voxel-level values are averaged over much larger and coarser anatomically-defined regions, affecting the performance of the classifiers. However, it is possible that differences between the voxel-based and region-based representation schemes will decrease if instead of an anatomical library, a functionally-derived segmentation method is used to determine the brain regions [38]. Moreover, it is interesting to note that differences in performance between the two representation schemes disappear in the across-participants classification results. Whereas a within-participant classifier must delineate a boundary that separates the scans in two classes based on the whole-brain pattern regularities of a single participant, across-participants classification must achieve the same by extracting regularities that are common to a group of individuals. The absence of differences between representation schemes in the across-participants classification results indicates that commonalities become much less evident when the training datasets contain data from several participants; when classifying whole-brain activity patterns of freely performed mental tasks, the linear SVM only capitalizes on the voxel-level information when dealing with data limited to a particular individual. Thus, to improve generalizability, across-participants classifiers should rely on data representations that abstract from the voxel-level data, such as measures of functional connectivity across different regions in the brain [38], [39].

Successful classification of brain imaging data, especially when employing data-driven machine learning techniques, does not necessarily imply that classification is driven by the activity of voxels that are relevant to the execution of the task in a physiological or cognitive sense. Because the SVMs transversally look at the activity of thousands of voxels at the same time, there is always a chance that classification will rely on whole-brain patterns of activity that are useful for discrimination purposes but are not meaningfully associated with the underlying cognitive processes of interest. We performed a GLM analysis using the same dataset that indicated distinct activity in regions that are broadly in line with past studies [26]–[29]. We then measured the similarity between the unthresholded statistical maps generated by the GLM analysis with the discriminating volumes generated by the SVMs, and found that the values are highly correlated. Taken at face value, these results indicate that the spatial distribution of the voxels driving the classification is consistent with the results of the GLM analysis, at least at the most basic level of changes in BOLD signal.

There are a few limitations of this study. First and foremost, minimum control was exerted during the performance of the tasks. Attention levels were not controlled within or across blocks of the same task type, nor they were equalized across tasks. Likewise, we did not control for difficulty across tasks, notably between the Negative and Positive Autobiographical Memory tasks, or the vividness and recency of the retrieved memories. This lack of uniformity could be especially critical when classifying scans across-participants. If the main purpose of this study had been solely the functional localization of brain areas involved in the execution of those tasks, under the lenses of conventional studies, such limitations would potentially disallow the interpretation of the results. However, in this study we deliberately chose a design where participants could perform the task under less constrained conditions, since the primary goal of the study was to address the question of whether it is possible to determine which mental task a participant is performing by examining whole-brain activity patterns that are evoked when one is more naturally engaged in executing a cognitive task. The underlying motivation is the view that experimental settings that attempt to finely control every possible aspect in play may be often grasping an incomplete view of the processes that take place in the real world [40]. That is likely to be especially true when examining the neural bases of higher-level cognitive-emotional processes.

Another limitation of this study is that, strictly speaking, there is no objective assessment that the participants were conforming to the instructions they were given, i.e., that they were executing the mental tasks they were asked to do. Results from the within-participant classification suggest that at the individual level, participants were at least performing distinct mental tasks at every other block. However, there are no guarantees that task execution was consistent across all participants, and that could be affecting the results of the across-participants classification.

The ability to classify single whole-brain scans extends the possibility of developing systems to communicate with patients who are fully conscious but unable to behaviorally respond to or interact with the external world [35], [36], [41], [42]. Real-time extensions of the current framework could be used in a fMRI-based conversational system [43], where answers are obtained from the patient on-site, and subsequent questions are defined based upon the answers. Sacrificing interpretability for the sake of improved classification accuracy, non-linear kernels such as the Gaussian radial basis function could be employed in place of the linear kernel used in the current study.

## Supporting Information

Table S1 Summary of some of the personal events recollected by the participants (P1–P11) during the Negative and Positive Autobiographical Memory mental tasks, reported at debriefing time.
(DOC)

File S1 Contains Figure S1.
(DOC)
