# Supplementary material for: Classification of Self-Driven Mental Tasks from Whole-Brain Activity Patterns
Source: PLoS One. 2014 May 13;9(5):e97296. doi: 10.1371/journal.pone.0097296 (PMC4019522; doi:10.1371/journal.pone.0097296)
Supplement: Table S1 — Summary of some of the personal events recollected by the participants (P1–P11) during the Negative and Positive Autobiographical Memory mental tasks, reported at debriefing time. (DOC) [file pone.0097296.s001.doc]

|  | Negative Autobiographical Memory | Positive Autobiographical Memory |
| --- | --- | --- |
| P1 | - *“The day I argued with the relatives of my spouse, the things they told me…”* - *“The day when our dog passed away. I was not present but I was told about the dog’s suffering…”* | - *“The day I met my friends, the things we laughed about…”* - *“When my baby was born, the first time I heard him crying, the relief I felt and the tears of joy…”* |
| P2 | - *“When I saw the girl I liked holding hands with my friend…”* | - *“The time I was traveling in ***, the great food I had there…”* |
| P3 | - *“When my pet of many years died…”* - *“When at the 18K mark I realized I wouldn’t finish the marathon.”* - *“The time I got involved in an accident.”* - *“The day I argued with a good friend.”* - *“When I tried snowboarding and failed…”* | - *“When I travelled with my friends.”* - *“When I was finally offered a job, and celebrated the occasion with my friends.”* - *“When my friends threw a surprise birthday party to me.”* |
| P4 | - *“The day I drank too much, and felt sick, my friends had to help me, had a bad hangover…”* | - *“When I went to ***, playing on the gorgeous beach resort, the excellent weather. The food was so great, and the landscape so moving…”* |
| P5 | - *“The last moments with my ill mother. All relatives came over to see her. The last words I exchanged with her…”* | - *“This time when I went out with my best friend, the things we talked, the great time we had…”* |
| P6 | - *“My time in elementary school, the problems I had with other students, when I refused to go, and felt insecure about the future…”* | - *“In the spring, watching the cherry trees fully blossomed, the sun shining, all elements making a great scenery. I felt so happy and fulfilled…”* |
| P7 | - *“In the summer a few years ago, when I was generally down, feeling angry, and people being angry at me…”* | - *“The great time I had in a trip to ***, the luxurious hotel room, and all the amusement we enjoyed…”* - *“When I went see a stage play with my favorite actor…”* |
| P8 | - *“This time I went touring with my bicycle far away, but got into some mechanical troubles…”* | - *“A day with a great weather, riding my bicycle to a pet shop, playing with the puppies there…”* - *“The time I went for a road trip with a friend…”* |
| P9 | - *“When I learned a friend had bad mouthed about me on my back…”* - *“When I got in trouble in my office…”* | - *“When I went out with my kids, they way they seemed to be so happy and enjoying themselves…”* - *“That time I was having my favorite dish, how happy I felt then…”* - *“When I went out to have fun with my friends…”* |
| P10 | - *“Watching my son’s soccer match, the way the coach was yelling at him…When he was substituted, the sadness on my son’s face…”* | - *“When I went for a trip with my family…”* - *“When I went to the beach with a childhood friend, played together in the sand, the dinner we had, and the laughs…”* |
| P11 | - *“When my bike was stolen. When I realized that it had been stolen… When I got involved in a road accident, being carried over right after it…”* | - *“Playing with my band on stage, when all the songs went great… Traveling with the band to ***, the thrill I used to feel on stage…”* |
