# Supplementary material for: Classification of Self-Driven Mental Tasks from Whole-Brain Activity Patterns
Source: PLoS One. 2014 May 13;9(5):e97296. doi: 10.1371/journal.pone.0097296 (PMC4019522; doi:10.1371/journal.pone.0097296)
Supplement: File S1 — Contains Figure S1. (DOC) [file pone.0097296.s002.doc]

Supporting Information

Classification of self-driven mental tasks from whole-brain activity patterns

N. E. Nawa & H. Ando

We performed additional analyses to address the question of whether data from a subset of brain areas can yield comparable results to the whole-brain based classification. We focused in the voxel level patterns in the inferior parietal lobule (bilaterally), because results from the GLM analysis showed that it was most consistently associated with the Countdown task, when compared to the Negative and Positive Autobiographical Memory tasks (Table 2). In order to avoid spurious “double-dipping” effects (even though, strictly speaking, this a posteriori analysis is not entirely bias-free because the choice for the inferior parietal lobule was based on results from the original GLM analysis, which used the whole dataset), we used half of the data (corresponding to the first 3 sessions involving the tasks) to perform a GLM analysis, with the same regressors of interest as the original analysis. Based on that, we computed the contrasts Countdown vs. Negative Autobiographical Memory (NAM) and Countdown vs. Positive Autobiographical Memory (PAM), and upon that, we applied spatial masks available in the Automated Anatomical Labeling to determine the surviving voxels in the inferior parietal lobule. The selected voxels were then used in the machine learning classification analysis performed in the remaining half of the data.

Using only half of the data reduced the power of the GLM analysis; the contrasts were thresholded at *p* < .005 (uncorrected; family-wise error and false-discovery error corrections proved to be too restrictive). We found 73 and 89 voxels meeting the criterion for the Countdown vs. NAM and Countdown vs. PAM, respectively. We then performed the exact same within-participant classification analysis, as we had done before using the voxel-based representation scheme, on the dataset limited to these voxels. Even though a few results still reached *p* < .05 significance (block-balanced permutation test) (see Figure 1 in this Supplementary Material), the overall classification accuracies were significantly lower than the results obtained using the whole-brain data set (Mann-Whitney-Wilcoxon test, *p* < .001), for both the Countdown vs. NAM (average accuracy across participants = 66.4%) and Countdown vs. PAM (average accuracy across participants = 59.9%).

These results indicate that the original classification results based on the whole-brain dataset are not being solely driven by patterns contained in the region most consistently associated with the Countdown task, namely, the inferior parietal lobule, suggesting that information distributed over voxels encompassing larger brain regions is determinant for successful classification.


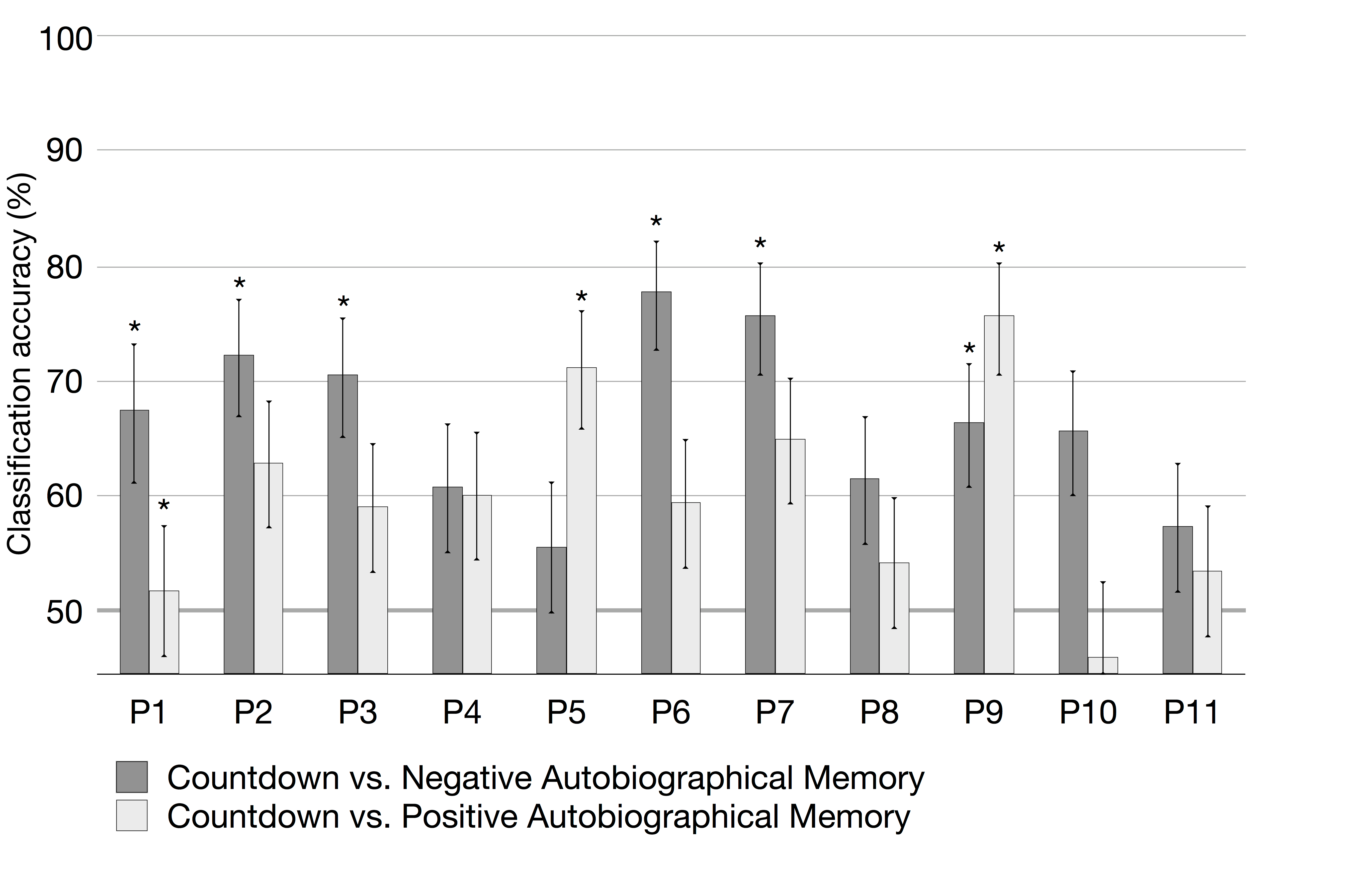


Figure S1: Within-participant classification accuracy in percent, using 73 (Countdown vs. NAM), and 89 (Countdown vs. PAM) voxels in the inferior parietal lobule (bilaterally) with linear SVMs, for each participant (P1–P11). Asterisks indicate *p <*.05 from a balanced-block permutation test, and the error bars are the 95% confidence intervals computed based on a beta distribution.
